# Cohort Profile: The Youth Vascular Consortium

**DOI:** 10.1007/s44200-026-00107-0

**Published:** 2026-03-06

**Authors:** Jun Young Park, Vimarsha Kodithuwakku, Alejandro Diaz, Christopher J. A. Pugh, Henner Hanssen, Manish D. Sinha, Emil Fraenkel, Hyeon Chang Kim, Rachel E. Climie

**Affiliations:** 1 Department of Preventive Medicine, Yonsei University College of Medicine, Yonsei University, 50-1 Yonsei-ro, Seodaemun-gu, Seoul, 03722 South Korea; 2 Menzies Institute for Medical Research, University of Tasmania, 17 Liverpool St, Hobart, TAS 7000 Australia; 3 Instituto de Investigación en Ciencias de la Salud, UNICEN-CCT CONICET, Tandil, Provincia de Buenos Aires Argentina; 4 Centre for Cardiovascular Research, Innovation and Development, Cardiff Metropolitan University, Cardiff, UK; 5 National Cardiovascular Research Network, Cardiff, Wales, UK; 6 Department of Sport, Exercise and Health, Medical Faculty, University of Basel, Basel, Switzerland; 7 Department of Paediatric Nephrology, Evelina London Children’s Hospital, Guy’s and St Thomas’ NHS Foundation Trust, London, UK; 8 British Heart Foundation Centre, King’s College London, London, UK; 9 Department of Internal Medicine, University of Košice, Košice, Slovakia; 10 Institue for Innovation in Digital Healthcare, Yonsei University Health System, Seoul, South Korea; 11 Université de Paris Cité, INSERM, U970, Paris Cardiovascular Research Center (PARCC), Paris, France

**Keywords:** Early vascular ageing, vascular health, youth, adolescence, cardiovascular risk, Youth Vascular Consortium

## Abstract

**Background:**

Cardiovascular disease remains the leading cause of mortality worldwide, yet its origins lie in early life. Cardiovascular risk factors track from childhood into adulthood, and vascular abnormalities detected in youth predict future cardiovascular outcomes. Despite compelling evidence, vascular assessment in youth has been impeded by a lack of reference values, standardised measurement protocols, and consensus on distinguishing physiological from pathological vascular ageing. The Youth Vascular Consortium (YVC) was established in 2020 to address these gaps through international collaboration between leading experts in the field.

**Results:**

The YVC comprises 33 research centers from 27 countries across five continents, including 29,704 participants aged 2 to 40 years. All centers assessed at least one validated vascular measure including pulse wave velocity, central blood pressure, augmentation index, intima-media thickness, carotid distensibility, or flow-mediated dilatation. The YVC has generated major outputs advancing vascular health assessment in youth, including international expert consensus on standardised definitions of early vascular ageing from birth through young adulthood and evidence-based recommendations for vascular assessment protocols. In addition, device-specific reference values for pulse wave velocity were established with age and sex specific percentile curves, enabling identification of youth with elevated arterial stiffness.

**Conclusions:**

The YVC provides an international platform for investigating vascular health from early life. Harmonising diverse datasets and establishing evidence-based standards, the Consortium aims to improve the vascular health of children and young people, thereby enabling early identification and targeted prevention strategies when vascular trajectories remain modifiable, ultimately reducing the global cardiovascular disease burden across the lifespan.

## Introduction

Cardiovascular disease remains the leading cause of mortality worldwide, yet its origins lie in early life [1]. Robust epidemiological evidence demonstrates that cardiovascular risk factors established during childhood and adolescence track persistently into adulthood, with individuals maintaining their relative risk profile within population distributions across decades [2–5]. Childhood risk factors exert both direct effects on adult cardiovascular disease and indirect effects mediated through the development of adult risk factors, establishing that early-life exposures shape lifelong disease trajectories [6–10]. The global burden of cardiovascular risk factors in youth has exacerbated markedly over recent decades, with dramatic increases in childhood obesity and paediatric hypertension documented worldwide [11–13]. These adverse trends demonstrate socioeconomic patterning and have translated into concerning patterns among young adults, where cardiovascular disease incidence and prevalence rates have increased or remained stable despite declining rates in older populations [1, 12, 14]. The convergence of worsening risk factor profiles in youth with evidence of strong tracking into adulthood portends substantial future increases in cardiovascular disease burden. Beyond the clinical impact, this trajectory poses a critical socio-economic threat on a global scale, driving escalating healthcare expenditures and long-term productivity losses that challenge the sustainability of health systems worldwide [15, 16].

The pathophysiological processes underlying cardiovascular disease begin during youth, with vascular abnormalities detectable and measurable before clinical disease manifestation. The concept of early vascular ageing characterises the dissociation between chronological and biological vascular age, whereby arterial structure and function deviate from age-expected norms [17–22]. Children and adolescents with obesity, hypertension, or metabolic abnormalities exhibit evidence of endothelial dysfunction, and measures of functional and structural arterial stiffening [23–30]. These vascular abnormalities associate with target organ injury and subclinical cardiac dysfunction [31], confirming that pathological processes progress actively during youth. Longitudinal studies have suggested that vascular abnormalities detected in youth predict future cardiovascular outcomes, with elevated pulse wave velocity and intima-media thickness in young adults predicting incident hypertension, atherosclerosis progression, and cardiovascular events [7, 32, 33]. Trajectories of cardiovascular health from childhood demonstrate that individuals maintaining optimal cardiovascular health profiles (blood pressure, cholesterol, glucose, body mass index, and healthy lifestyle behaviors) experience substantially lower vascular damage in middle age, with this association persisting after adjustment for traditional risk factors [7].

Recognition of these critical developmental processes has led to growing consensus that vascular assessment in youth offers important opportunities for cardiovascular disease prevention. International expert groups have emphasized the need for lifetime cardiovascular risk assessment beginning early in life, acknowledging that disease development reflects cumulative risk exposure combined with individual susceptibility [34]. The concept of early vascular ageing has gained prominence as a framework for identifying youth at elevated risk, with measures of arterial stiffening such as pulse wave velocity, and vascular structure measurements proposed as tools to capture subclinical disease progression beyond traditional risk factor assessment [17–19, 22]. However, translation of these concepts into clinical practice has been limited by fundamental gaps in knowledge and methodology. Furthermore, the high cost of specialized instrumentation required for accurate vascular assessment remains a significant barrier to widespread clinical implementation, particularly in resource-limited settings [15, 35].

Despite compelling evidence linking early-life vascular abnormalities to future cardiovascular outcomes, significant barriers have impeded routine vascular assessment in young populations. Reference values spanning infancy through young adulthood have been lacking, with existing studies limited by small samples, narrow age ranges, and geographic homogeneity. Standardised measurement protocols specific to youth have been absent, with guidelines typically extrapolated from adult studies without developmental considerations. The optimal biomarkers for identifying early vascular ageing at different life stages remain uncertain, as do thresholds distinguishing physiological from pathological vascular ageing. Device-specific measurement differences have introduced additional complexity, as commercially available systems employ distinct methodologies, potentially limiting direct comparability. These methodological challenges have prevented widespread clinical adoption of vascular assessment in youth despite growing recognition of its potential value.

The Youth Vascular Consortium (YVC) was established to address these critical gaps through international collaboration and systematic data harmonisation [36]. By pooling diverse cohorts spanning multiple continents, age groups, and methodological approaches, the Consortium has generated standardised definitions of early vascular ageing, established measurement protocols and quality standards, and developed device-specific reference values enabling clinical interpretation of vascular measurements [37–39]. The primary aim of the YVC is to establish age‑ and sex‑specific reference intervals for widely used non‑invasive vascular ageing biomarkers in healthy individuals aged 0–40 years. A secondary aim is to compare vascular ageing biomarkers directly and assess their relationships with ageing, growth, and cardiometabolic risk. The present cohort profile describes the Consortium structure, methodology, and initial findings, presenting characteristics of participating centers, harmonisation strategies employed, key outputs generated, and strengths and limitations of this international collaborative approach.

## Methods

### Study Design and Participant Recruitment

The YVC is a multicentre collaborative study established to investigate vascular ageing in youth through international data harmonisation [36]. Research groups with existing datasets containing vascular measurements in individuals aged 0 to 40 years were identified through literature review and professional networks and invited to participate. This age threshold was defined based on established research frameworks that identify the first four decades of life as a critical window for primordial prevention, before the typical onset of overt clinical cardiovascular events [40, 41].

Eligibility criteria required appropriate ethical approval with explicit participant consent for future research use, collection of basic demographic and anthropometric data, and assessment of at least one validated vascular measure. Participating investigators submitted detailed study documentation, including research protocols, ethical approvals, and participant consent forms. Data collection for the Consortium is ongoing and the Consortium remains open to continuous expansion as additional eligible centers are identified or are able to meet participation requirements.

### Data Collection and Harmonisation

Participating collaborators provided standardised datasets according to Consortium templates specifying essential and non-essential variables. Essential variables included age, sex, ethnicity, height, weight, brachial blood pressure, and at least one vascular measurement, including pulse wave velocity, central blood pressure, augmentation index, intima-media thickness, carotid distensibility, or flow-mediated dilatation. Non-essential variables included additional biochemical markers, lifestyle factors, and socioeconomic indicators. For each vascular measurement, investigators documented comprehensive methodological details including device specifications, software versions, measurement sites, participant preparation procedures, environmental conditions, and quality control approaches.

Data harmonisation addressed multiple sources of heterogeneity across contributing centers. For pulse wave velocity, documentation included path length calculation methods, pulse wave detection algorithms, and number of replicate measurements. For repeated pulse wave velocity measurements, the final measurement used for analysis was determined in accordance with the European Network for Noninvasive Investigation of Large Arteries guidelines [42]. Central blood pressure values were combined according to the standardized procedure [43]. Blood pressure measurement protocols specified device types, participant position, rest duration, and averaging procedures. For intima-media thickness, protocols detailed arterial segments assessed, image acquisition techniques, edge detection methods, and number of measurements. Quality control procedures included verification of physiologically plausible value ranges, identification of outliers through graphical and statistical methods, and consistency checks between related variables.

## Results

### Participating Centers and Study Population

**Fig. 1:**
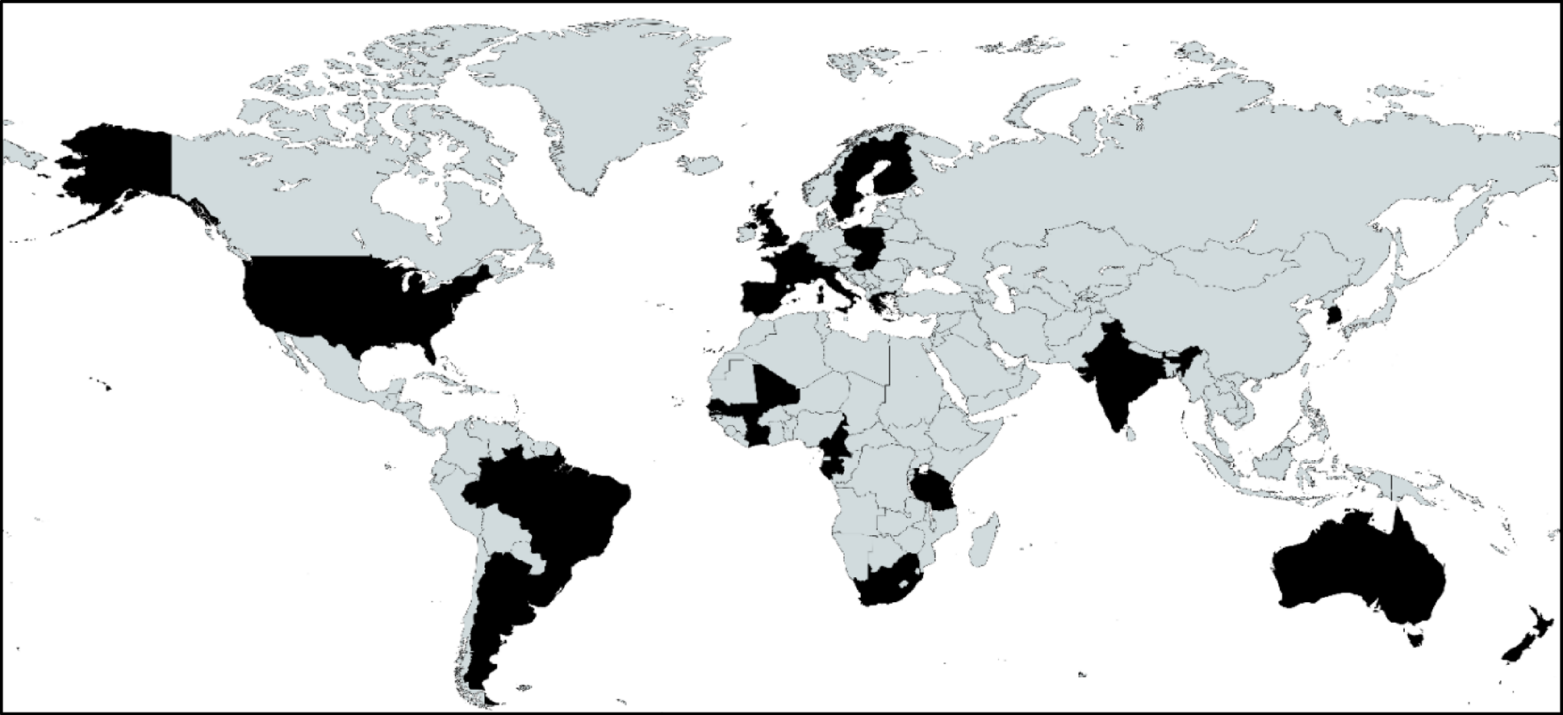
Countries in the Youth Vascular Consortium. Data from the countries shaded in black are included in Consortium as of February 2026 (*n* = 27 countries)

**Fig. 2:**
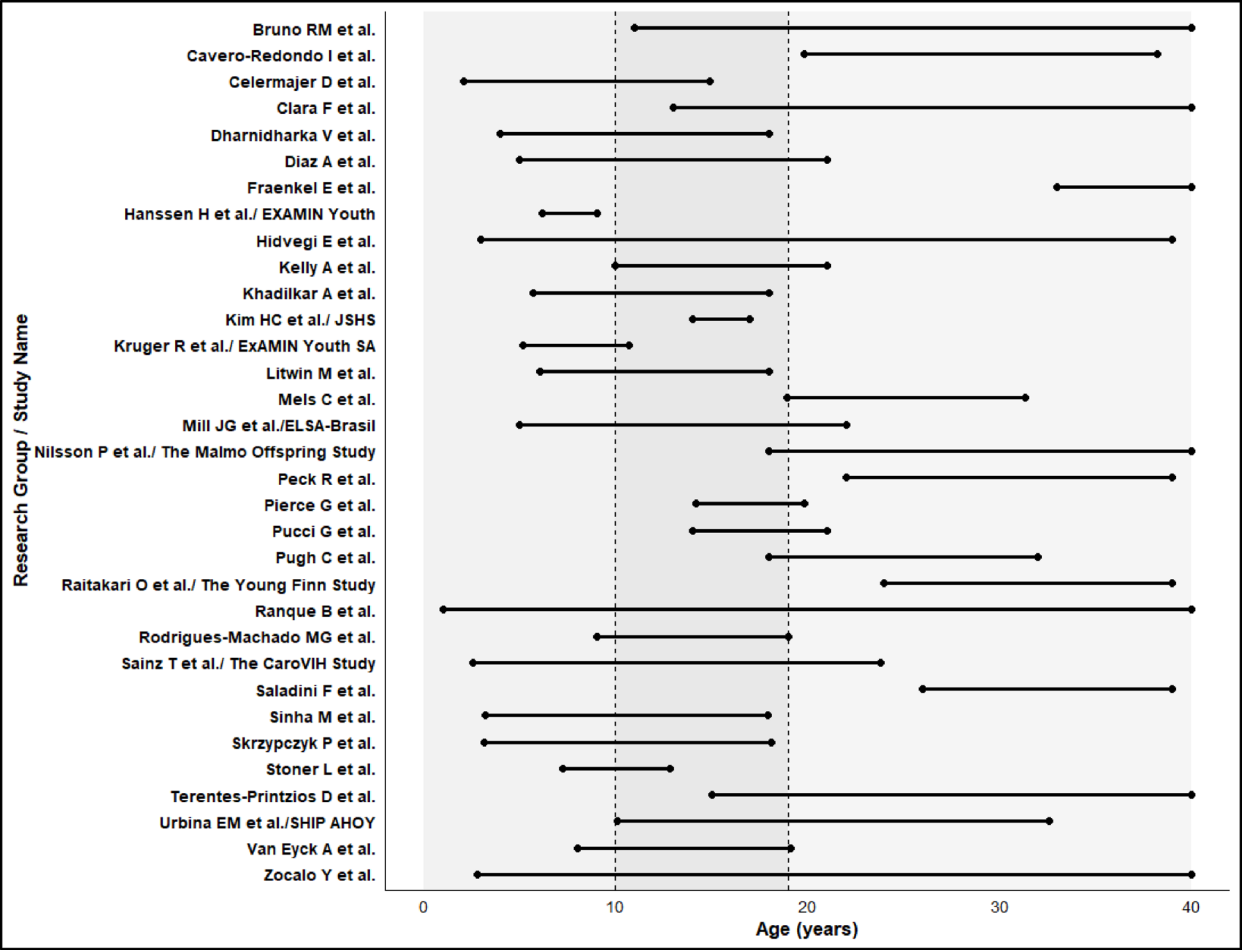
Age distribution of cohorts included in the Youth Vascular Consortium. The shaded areas represent childhood, adolescence and young adulthood. Adolescence was defined based on the World Health Organization’s definition (area in the middle of the dashed lines)

Table 1 summarises characteristics of the 33 research centers comprising the YVC. Studies were conducted in 27 countries spanning Europe (12 countries), North and South America (4 countries), Africa (6 countries plus one unspecified region), Asia (2 countries), and Oceania (2 countries), collectively enrolling 29,704 participants (Fig. 1). As of February 2026, we have invited 239 research centers to participate. Some centers were unresponsive (20%) and many (67%) were unable to participate currently due to practical constraints including ethics approvals for their data to be used for future use and data‑transfer requirements. These constraints are unlikely to reflect systematic or scientific differences between participating and non-participating cohorts. Study sample sizes ranged from 12 to 7,622 participants. Specifically, 19 centers enrolled fewer than 500 participants, 9 centers enrolled 500–1,499 participants, and 5 centers enrolled 1,500 or more participants. Study conduct periods spanned 1980 to 2023, with 24 studies initiated after 2005. Participant age at baseline ranged from 2 to 40 years across studies, with 14 studies enrolling predominantly children and adolescents under 18 years, 8 studies focusing on young adults over 18 years, and 11 studies spanning both paediatric and adult age ranges. Sex distribution was balanced overall, with individual studies ranging from 15 to 83% male participants. Figure 2 illustrates the age distribution of participants across the Consortium, demonstrating coverage across the developmental continuum from early childhood through young adulthood.

**Table 1:** Summary of cohorts included in Youth Vascular Consortium

Research Center/Study Name	Country	Total participants	Conducted year	Age range (years)	Male sex (%)
Bruno RM et al.	France	1310	1995 to 2005	11–40	50
Cavero-Redondo I et al.	Spain	67	2017	20–39	30
Celermajer D et al.	Australia	193	2013 to 2013	2–15	55
Clara F et al.	Argentina	262	2004 to 2023	13–40	49
Dharnidharka V et al.	USA	95	2014 to 2014	4–18	54
Diaz A et al.	Argentina	1038	2015 to 2015	5–21	56
Fraenkel E et al.	Slovakia	15	2023	33–40	45
Hanssen H et al./EXAMIN Youth	Switzerland	1463	2016 to 2017	6–9	49
Hidvegi E et al.	Hungary	7622	2008 to 2023	3–38	54
Kelly A et al.	USA	249	2015 to 2016	10–21	43
Khadilkar A et al.	India	201	2008 to 2009	6–18	48
Kim HC et al./JSHS Study	South Korea	1070	2007 to 2012	14–17	52
Kruger R et al./ExAMIN Youth SA	South Africa	1064	2015 to 2017	5–11	46
Litwin M et al.	Poland	339	2012 to 2020	6–18	79
Mels C et al.	South Africa	1263	2016	19–31	48
Mill JG et al./ELSA-Brasil	Brazil	1744	2018	5–22	51
Nilsson P et al./The Malmö Offspring Study	Sweden	1482	2016 to 2016	18–40	48
Peck R et al.	Tanzania	13	2019 to 2020	22–39	15
Pierce G et al.	USA	227	2009 to 2010	14–20	53
Pucci G et al.	Italy	528	2015 to 2015	14–21	60
Pugh C et al.	UK	220	2013 to 2018	18–32	78
Raitakari O et al./Young Finn	Finland	2434	1980 to 2007	24–39	49
Ranque B et al.	Cameroon, Senegal, Ivory Coast, Mali, and Gabon	792	2011 to 2013	1–40	41
Rodrigues-Machado MG et al.	Brazil	132	2016 to 2016	9–19	50
Sainz T et al./The CaroVIH Study	Spain	312	2011	3–24	39
Saladini F et al.	Italy	12	2001	26–39	83
Sinha M et al.	UK	246	2011 to 2012	3–18	58
Skrzypczyk P et al.	Poland	374	2018	3–18	61
Stoner L et al.	New Zealand	392	2015 to 2016	7–13	50
Terentes-Printzios D et al.	Greece	149	2005 to 2005	15–40	73
Urbina EM et al./SHIP AHOY	USA	1967	2008 to 2016	10–33	44
Van Eyck A et al.	Belgium	281	2018 to 2021	8–19	43
Zocalo Y et al.	Uruguay	2148	2015 to 2017	3–40	49

### Availability of Measurements

Table 2 summarises the availability of core demographic, anthropometric, and vascular measurements across the 33 research centers participating in the YVC. Sex and age were recorded in all studies, while ethnicity was reported in 26 studies. Date of measurement was documented in 29 studies, reflecting heterogeneity in study timing and design. Anthropometric measurements were widely collected. Body mass index was collected at all 33 centers. Measures of body fat distribution were less consistently reported, with waist circumference available from 14 centers and hip circumference from 11.

**Table 2:** Core data provided by the research centers included in the Youth Vascular Consortium

Research Center/Study Name	Demographics				Anthropometrics					Vascular measurements						Other haemodynamic measurements			
	Sex	Age	Ethnicity	Date of measurement	Height	Weight	BMI	Waist circumference	Hip circumference	Blood pressure—Brachial	Blood pressure—Central	Pulse wave velocity	Reactive hyperemia index	Carotid intima-media thickness	Flow-mediated dilatation	Augmentation index	Cardiac output	Stroke volume	Peripheral resistance
Bruno RM et al.	✔	✔	-	✔	✔	✔	✔	-	-	✔	-	✔	-	-	-	-	-	-	-
Cavero-Redondo I et al.	✔	✔	✔	✔	✔	✔	✔	✔	-	✔	-	✔	-	✔	-	-	-	-	-
Celermajer D et al.	✔	✔	✔	✔	✔	✔	✔	✔	✔	✔	-	✔	-	✔	✔	-	-	-	-
Clara F et al.	✔	✔	-	-	✔	✔	✔	-	-	✔	-	✔	-	-	-	✔	-	-	-
Dharnidharka V et al.	✔	✔	✔	✔	✔	✔	✔	-	-	✔	-	✔	-	-	-	✔	-	-	-
Diaz A et al.	✔	✔	✔	✔	✔	✔	✔	-	-	✔	✔	✔	-	-	-	-	-	-	-
Fraenkel E et al.	✔	✔	-	-	-	-	✔	-	-	✔	-	-	-	-	-	✔	-	-	-
Hanssen H et al./EXAMIN Youth	✔	✔	-	✔	✔	✔	✔	-	-	✔	✔	✔	-	-	-	-	-	-	-
Hidvegi E et al.	✔	✔	✔	✔	✔	✔	✔	-	-	✔	✔	✔	-	-	-	-	-	-	-
Kelly A et al.	✔	✔	✔	✔	✔	✔	✔	✔	✔	✔	✔	✔	-	-	-	-	-	-	-
Khadilkar A et al.	✔	✔	✔	✔	✔	✔	✔	✔	✔	✔	-	✔	-	-	-	-	-	-	-
Kim HC et al./JSHS Study	✔	✔	✔	✔	✔	✔	✔	-	-	✔	-	-	-	✔	-	-	-	-	-
Kruger R et al./ExAMIN Youth SA	✔	✔	✔	✔	✔	✔	✔	✔	-	✔	✔	✔	-	-	-	-	-	-	-
Litwin M et al.	✔	✔	✔	✔	✔	✔	✔	✔	✔	✔	✔	✔	-	-	-	-	-	-	-
Mels C et al.	✔	✔	✔	✔	✔	✔	✔	-	-	✔	✔	✔	-	-	-	-	-	-	-
Mill JG et al./ELSA-Brasil	✔	✔	✔	✔	✔	✔	✔	✔	✔	✔	✔	✔	-	-	-	✔	-	-	-
Nilsson P et al./The Malmö Offspring Study	✔	✔	✔	✔	✔	✔	✔	-	-	✔	✔	✔	-	-	-	✔	-	-	-
Peck R et al.	✔	✔	-	✔	✔	✔	✔	-	-	✔	-	✔	-	-	-	-	-	-	-
Pierce G et al.	✔	✔	✔	-	✔	✔	✔	✔	✔	✔	✔	✔	-	-	-	✔	-	-	-
Pucci G et al.	✔	✔	✔	✔	✔	✔	✔	-	-	✔	✔	✔	-	✔	-	-	-	-	-
Pugh C et al.	✔	✔	✔	✔	✔	✔	✔	-	-	✔	✔	✔	-	-	-	✔	-	-	-
Raitakari O et al./Young Finn	✔	✔	✔	✔	✔	✔	✔	-	-	✔	-	✔	-	-	-	-	-	-	-
Ranque B et al.	✔	✔	-	✔	✔	✔	✔	-	-	✔	✔	✔	-	-	-	✔	-	-	-
Rodrigues-Machado MG et al.	✔	✔	-	✔	✔	✔	✔	✔	✔	✔	✔	✔	-	-	-	✔	-	-	-
Sainz T et al./The CaroVIH Study	✔	✔	✔	-	✔	✔	✔	✔	✔	✔	-	-	-	✔	-	-	-	-	-
Saladini F et al.	✔	✔	✔	✔	✔	✔	✔	-	-	✔	✔	✔	-	-	-	-	-	-	-
Sinha M et al.	✔	✔	✔	✔	✔	✔	✔	-	-	✔	✔	✔	-	-	-	-	-	-	-
Skrzypczyk P et al.	✔	✔	✔	✔	✔	✔	✔	✔	✔	✔	✔	✔	-	✔	-	✔	-	-	-
Stoner L et al.	✔	✔	✔	✔	✔	✔	✔	✔	✔	✔	✔	-	-	-	-	-	-	-	-
Terentes-Printzios D et al.	✔	✔	✔	✔	✔	✔	✔	-	-	✔	✔	✔	-	-	✔	-	-	-	-
Urbina EM et al./SHIP AHOY	✔	✔	✔	✔	✔	✔	✔	✔	-	✔	✔	✔	-	✔	✔	-	-	-	-
Van Eyck A et al.	✔	✔	✔	✔	✔	✔	✔	✔	✔	✔	-	-	✔	-	-	✔	-	-	-
Zocalo Y et al.	✔	✔	✔	✔	✔	✔	✔	-	-	✔	✔	✔	-	✔	✔	-	✔	✔	✔

Vascular phenotyping constituted the central focus of data collection. Brachial blood pressure was measured in all 33 centers, whereas central blood pressure measurements were available in 21. Pulse wave velocity, the primary marker of arterial stiffness, was reported by 28 of the 33 research centers. Additional vascular measures were available in a smaller number of cohorts. Carotid intima-media thickness was measured in 8 centers, flow-mediated dilatation in 4, reactive hyperaemia index in 1, and augmentation index in 11.

## Discussion

The YVC comprises 29,704 participants from 33 research centers across 27 countries, providing an international platform to investigate vascular ageing in youth. This cohort profile describes the Consortium structure, participating studies, available measurements, and harmonisation approaches. The geographic and ethnic diversity, coupled with comprehensive vascular phenotyping across ages 2 to 40 years, enables investigation of vascular development trajectories and identification of early vascular ageing across diverse populations. The primary achievement of the YVC lies in its ability to repurpose high-quality datasets that might otherwise remain underutilised within individual centers. By pooling these disparate data, the Consortium has not only extended the utility of existing research beyond its original scope but has also catalysed the generation of new knowledge and ideas that would be unlikely to be achieved individually.

### Major Findings and Outputs to Date

The Consortium has produced major outputs advancing vascular health assessment in youth. An international expert consensus established standardised definitions of early vascular ageing applicable from birth through young adulthood, identifying biological and environmental factors associated with accelerated vascular ageing at different developmental stages [37]. Complementary methodological consensus work reviewed assessment methods for early vascular ageing in youth and the gaps in current methods [38]. Moreover, device-specific reference values for pulse wave velocity were established using 19,930 healthy participants, with percentile curves stratified by age, sex, and device type enabling identification of youth with elevated arterial stiffness [39]. Reference values were provided for six measurement systems including SphygmoCor, Complior, Vicorder, Arteriograph, Mobil-O-Graph, and Pulsepen. Ongoing work is developing reference values for other biomarkers of vascular ageing in youth.

### Strengths and Limitations

Key strengths include a large sample size enabling adequately powered analyses, geographic and ethnic diversity enhancing generalisability, comprehensive vascular phenotyping permitting comparative biomarker analyses, and systematic harmonisation strategies despite methodological heterogeneity. The collaborative structure facilitates ongoing expansion and longitudinal follow-up. Important limitations include retrospective design with potential selection bias, incomplete biochemical characterisation with limited metabolic marker availability, inconsistent lifestyle and socioeconomic data collection constraining behavioural and social determinant analyses, uneven geographic representation with European predominance and limited African and Asian participation, device heterogeneity requiring device-specific analyses, and limited longitudinal data precluding within-individual trajectory examination for most cohorts.

### Future Directions

Ongoing consensus work by the Consortium will determine the clinical applicability of vascular measures in youth and will provide measurement guidelines for various vascular measures in youth. While the focus to date has primarily been on pulse wave velocity, ongoping work aims to increase the depth of data within the Consortium to include additional vascular measures, subclinical outcome data, and other factors such as smoking status, lifestyle behaviors, socioeconomic status, and maturation status. Furthermore, the integration of longitudinal data will be a key priority for the Consortium in the coming years. These efforts aim to create a more comprehensive framework for understanding vascular health trajectories in youth.

## Conclusion

Cardiovascular disease prevention requires action beginning in early life, when pathological vascular processes initiate, and risk factor trajectories are established. The YVC addresses this imperative by providing the infrastructure, standards, and evidence base necessary to translate vascular assessment into clinical practice for youth populations. Through international collaboration harmonising diverse datasets, the Consortium has established standardised definitions of early vascular ageing and generated device-specific reference values enabling identification of youth with accelerated vascular ageing. The collaborative infrastructure provides a platform for investigations of ethnic and regional differences in vascular trajectories, socioeconomic and lifestyle determinants, and validation of vascular biomarkers for cardiovascular risk prediction. By welcoming additional centers from underrepresented regions, supporting the development of reference values for additional vascular parameters and inclusion of longitudinal data, the Consortium continues to expand its scope and impact. We believe our collaborative approach to understanding vascular health in youth will enable early identification of at-risk individuals and facilitate targeted interventions during developmental periods when vascular trajectories remain modifiable, ultimately reducing the global burden of cardiovascular disease across the lifespan.

## Data Availability

Data from the Consortium are available for collaborative research upon approval by the YVC Research Committee. Researchers interested in accessing data should complete a Proposed Research Project Form and submit it to the Research Lead ([rachel.climie@utas.edu.au] (mailto: rachel.climie@utas.edu.au)). Information about Consortium participation and available data parameters can be found at [https://www.youthvascularconsortium.com/] (https:/www.youthvascularconsortium.com).
